# Balance matters more: Research on the effect of corporate social responsibility equilibrium on stock price crash risk

**DOI:** 10.1371/journal.pone.0306879

**Published:** 2024-10-03

**Authors:** Siyuan Yu, Ming Tian

**Affiliations:** 1 Institute for Innovation and Entrepreneurship, Nanjing Vocational University of Industry Technology, Nanjing, Jiangsu, China; 2 Business School, Hohai University, Nanjing, Jiangsu, China; 3 World Water Valley Institute, Nanjing, Jiangsu, China; Nanjing Audit University, CHINA

## Abstract

The impact of the degree and effectiveness corporate social responsibility (CSR) fulfillment on corporate value assessment by investors is significant. However, prior research on effects of CSR on stock price crash risk has showed contrasting results. Certain studies suggest that an abundance of CSR can become a self-serving tool for management. This may lead to concealing and accumulating negative information, resulting in stock price collapse. Based on principal-agent theory, we suggest that CSR equilibrium could be an effective approach to prevent the concealment of negative events by managers. Employing data from Chinese A-share listed companies spanning 2010 to 2020, we examine how CSR equilibrium and corporate governance levels influence the risk of stock price collapse across entities with different property rights. Empirical findings indicate that a balanced distribution of CSR can mitigate the risk of stock price collapse. Furthermore, we find that higher levels of corporate governance can mitigate the negative impact of CSR equilibrium on stock price collapse risk. Interestingly, this governance effect is more pronounced in state-owned enterprises compared to private enterprises, where the likelihood of hiding adverse information is higher. Additionally, it provides a theoretical explanation for the impact of CSR equilibrium on the risk of stock price collapse, based on the principal-agent problem, thereby expanding the applicability of the theory. Practically, the research findings carry significant implications for listed companies, CSR rating agencies, and investors.

## Introduction

Recently, as the concept of sustainable development has gained widespread acceptance, various sectors of society have consistently focused on assessing the extent and effectiveness of corporate social responsibility (CSR). Investors consider a company’s CSR performance as a crucial criterion in evaluating its management quality [1]. Despite some companies being well-regarded for their high CSR standards, they have been found engaging in harmful practices like financial fraud, food safety breaches, and environmental harm [2]. The unexpected disclosure of such negative information often results in persistent declines in stock prices. These events have prompted scholars to reflect on whether CSR levels can serve as a basis for predicting the risk of stock price collapse, thereby reflecting the company’s governance objectives [3].

Existing research on the relationship between CSR and stock price crash risks has primarily focused on issues stemming from information asymmetry and principal-agent problems [4]. While, these studies have yielded mixing empirical results suggesting that theoretically CSR could have both positive and negative effects on stock price crash risk, and some on information disclosure indicating that proactive CSR can increase the transparency in company information, thereby reducing information asymmetry and lowering the risk of stock price collapse [5]. Conversely, there are studies arguing that CSR might be used by managers as a self-serving tool to hide negative information from the public, potentially increasing the risk of stock price collapse [6]. Managers who consider fulfilling social responsibilities as a "self-interest tool" may blindly invest in dimensions such as charity without considering the cost without considering the cost, leading to situations where they publicly practice philanthropy while simultaneously harming the interests of employees and consumers, known as "dichotomy convergence" [7]. Such hypocritical behavior could potentially impact on stock price collapse.

Conflicting conclusions in existing studies arise partly from a focus on examining the extent of CSR performance while neglecting the issue of its effectiveness. The above contradictory empirical conclusions reveal a research gap that is except for the level of CSR performance what other CSR indicator could affect companies’ stock price collapse risk from an agency theory perspective. In order to bridge the research gap, this study focuses on the effectiveness of CSR by examining the concept of CSR equilibrium. By emphasizing the balance of interests and safeguarding the rights of all stakeholders, CSR equilibrium helps prevent CSR from deviating from its original intention, or becoming a sacrifice of agency conflicts [8]. However, as a relatively new research topic, limited research exists on the impact of CSR equilibrium on the risk of stock price collapse. Focusing solely on the level of CSR performance not only leads to misunderstandings about the relationship between CSR and stock price collapse risk but also hinders the implementation of specific social responsibility practices by companies. Therefore, this study examines the influence of CSR balance on the risk of stock price collapse from the perspective of CSR equilibrium, providing a framework for the stability of company stock prices.

Furthermore, studies explaining the stock price crash problem from a governance theory perspective find that corporate governance can effectively mitigate the crash risk. Inappropriate corporate governance structures could incentivize management teams to prioritize short-term financial gains, and deviating from the original intention of fulfilling social responsibility. Conversely, better corporate governance mechanisms can reconcile conflicts between stakeholders and reduce problems such as stock price collapse caused by conflicting interests [9–11]. This study aims to delve deeper into the influence of corporate governance on CSR equilibrium in relation to the risk of stock price collapse.

The main theoretical contributions of this study are two-fold. Firstly, it enriches the existing research on the impact of CSR on stock price collapse by adopting the perspective of social responsibility balance to explain the antecedent variables affecting stock price collapses. Secondly, based on governance theory, this study explores how f CSR equilibrium affects the risk of stock price collapse by verifying the contingent effects of corporate governance level and corporate ownership structures. From a practical aspect, findings of this study can help investors identify the company’s performance motives through CSR activities more accurately, thereby making more precise assessment about the investment risk of the company. This has important implications for reducing the risk of stock price collapse and protecting the legitimate rights and interests of stakeholders.

The subsequent sections of this study are organized as follows: “Literature review and hypothesis development” shows the definition of CSR equilibrium used in this study, past literature on agency theory, and outlines hypothesis development. ’Research design’ details data collection methods and hypothesis testing. The study then presents empirical results, discusses contributions and limitations.

## Literature review and hypothesis development

### Corporate social responsibility equilibrium

CSR encompasses a wide set of organizational obligations [12] that beyond companies’ direct economic objects [13]. Traditional studies Adopting a static perspective classic study categorize CSR into four levels, namely economic responsibility, legal responsibility, ethical responsibility, and philanthropic responsibility [14]. However, a dynamic perspective, as emphasized in recent theoretical research, focuses on the equilibrium of CSR [8, 15]. The concept of equilibrium has two theoretical interpretations: equality of variables where indicators of various dimensions are quantitatively equal, and a state of balance where stakeholders are satisfied with the current state and lack the motivation or ability to change it [16, 17]. In this study, the theoretical meaning of CSR equilibrium refers to the balanced behavior of enterprises in fulfilling complex social contracts. It represents the relatively close levels of fulfillment towards shareholders, employees, stakeholders, the environment, and philanthropy, and the coordination and equilibrium of the fulfillment levels among these dimensions.

The main reason why CSR equilibrium is interpreted as balanced CSR praxis in this study was determined by research focus. Previous studies have mostly focused on how enterprises maximize their interests in fulfilling CSR, and even how management, in order to avoid short-term performance evaluation pressure and legal risks, may lack the motivation to fulfill social responsibilities [18]. This study, however, focuses on the relationship between the different levels of CSR and stock price crash risk emphasizing how to maximizing corporate interests through multi-party bargaining [14, 15] Therefore, the emphasis of CSR equilibrium in this study is on enterprises equally fulfilling different types of social responsibility, rather than solely striving to fulfill certain core aspect of CSR.

Existing research has argued that companies actively practice CSR to gain legitimacy [19, 20]. Legitimate acquisition does not necessarily require extensive organizational resources to participate in multidimensional CSR. Consequently, companies may selectively fulfill their social responsibilities without addressing all types of social responsibilities equally [15]. Such selective CSR practices are often seen as peripheral, collateral, and driven by utilitarian motives. One dominant orientation behind selective CSR practices is the focus on market logic and the desire to economic value [16]. Failure to implement CSR in a balanced manner is detrimental to the long-term stability of the enterprise and tends to increase the risk of a share price crash [8]. Viewing a balanced approach to social responsibility as a strategic corporate initiative prevents CSR from being reduced to a self-interested tool for management [16, 21].

Other scholars focusing on CSR investment value the economic consequences of social responsibility fulfillment more than other aspects. Some researchers argue that selective fulfillment of CSR conveys a responsible corporate image to the outside world while avoiding excessive investment of resources in social responsibility [22]. Additionally, literature suggests that an balanced approach to CSR may hinder stakeholders’ perception and fail to contribute to overall performance improvement [15]. Relevant studies also highlighted that the balanced or non-balanced fulfillment of CSR is based on whether it is possible to obtain a return on the transactions of stakeholders and increase the competitive resources of the company [23].

This study understands CSR equilibrium as the harmonious fulfillment of a complex social contract involving shareholders, suppliers, and the environment. Given the close relationship between the principal-agent problem and the risk of stock price collapse, this study will further explore the impact of CSR equilibrium on stock price crash risk based on principal-agent theory.

### Principle- agency theory

Principle-agent theory serves as the theoretical framework for generating hypotheses in this study. The risk of stock price collapse, as perceived by investors, is attributed to the information asymmetry between market investors and corporate managers. This information gap results in varying expectations regarding the company’s stock price fluctuations among different stakeholders [24, 25]. Additionally, it increases agency costs in various ways [26]. This information asymmetry is the logical starting point of principal-agent theory. Existing research on the relationship between CSR performance and stock price crash risk also points out that the quantity and quality of CSR disclosure will lead to an information gap between investors and corporate managers [27]. This difference generates Ex post moral hazard and raise agency costs [28].

The principal-agent relationship is a set of both explicit and implicit contracts between shareholders and management [27]. Agents who manage the company provide services to various principals based on agreements to serve the principals’ interests, while also receiving economic returns upon completing assigned tasks [29]. Due to the necessity of perfecting contracts, stakeholders in an enterprise often have diverging interests, leading to potential conflicts. This may result in stakeholders prioritizing their own interests over others’, sometimes even at the expense of other entities [29]. Therefore, agency theory serves as a comprehensive framework for understanding and improving contractual relationships.

According to the principal-agent theory, the disclosure of social responsibility information may serve as a tool for whitewashing, allowing managers to potentially conceal unethical behavior like hiding poor profitability or other negative news, and shifting the focus of shareholders [30]. For example, Lin et al. (2015) suggests that Chinese companies can leverage social responsibility initiatives to establish political connections, which can benefit the company [31]. Therefore, the intricate connection between CSR and the potential risk of a decline in its stock price warrants further investigation.

### Corporate social responsibility equilibrium and stock price collapse

Research on stock price crash potential has predominantly focused on the influence of principal-agent dynamics and information transparency arising from CSR practices. Proponents of a positive perspective argue that companies with a strong commitment to social responsibility are more likely to uphold higher ethical standards leading to lower agency costs and reduce information asymmetry [18]. Consequently, such companies could reduce stock price collapse possibility through the "communication effect" [27]. Conversely, critics contend that CSR implementation may serve as a mere facade to conceal unethical practices.

Derived from the managerialist view based on principal-agent theory, the fulfillment of CSR may serve management’s self-interest more than shareholders’ interests [32]. Managers may selectively fulfill certain CSR aspects based on their personal values, characteristics, or desires to cover up scandals [33]. For instance, managers with religious beliefs might prioritize charitable responsibilities over employee, product, or environmental responsibilities [34]. Female executives might pay more attention to environmental responsibilities [35], while second-generation managers of family firms may focus on internal responsibilities to shareholders and employees after taking over [36]. Imbalances in CSR fulfillment can increase risks in specific dimensions of responsibility, reduce corporate transparency and heighten the risk of stock price collapse. Although some enterprises may gain a competitive advantage edge by fulfilling a single dimension of CSR, this approach often leads to excessive marketing and CSR hype [3]. During times of drastic external changes, such enterprises may face rapid decline or failure when encountering unexpected crisis events.

In contrast, equilibrious CSR implies balancing and mutually supervising stakeholders. Due to the principal-agent relationship, investors do not directly engage in the operational decision-making or management evaluation of the company. They rely on information to evaluate the impact of the company’s social responsibility performance on stock price [8, 37]. With the growing acceptance of responsible investment by ordinary investors, analysts are increasingly focusing on the balanced fulfillment of CSR. Institutional investors have observed that balanced CSR fulfillment could enhance the transparency of corporate operating activities when actively performing their supervisory role [38]. Higher information transparency reduces the agency costs that management may incur in fulfilling CSR, thereby decreasing the risk of stock price collapse. For example, Hsueh & Chang (2008) argue that in a supply chain network, the more balanced the CSR collaboration among various members is, the higher the total profit of each member enterprise will be [23]. The balanced practice of CSR, to some extent, indicates that corporate managers adhere higher ethical standards. This indicates potentially reducing earnings management, enhancing the quality of financial information, and decreasing the likelihood of managers deliberately concealing "bad news." To a certain degree, it mitigates the potential problem of adverse selection and moral hazards. By balancing responsibilities, it can alleviate information asymmetry between companies and investors, assisting investors in making effective investments and avoiding adverse impacts and uncertainties in corporate operations. Therefore, we propose the following hypothesis:

H1: the higher degree of CSR equilibrium is, the lower risk of companies’ stock price crash will be.

### The moderating effect of company’s governance level between CSR equilibrium and stock price collapse

Corporate governance functions as a mechanism for aligning stakeholders’ interests. In conjunction with CSR equilibrium, it helps alleviate the agency problem by reducing information asymmetry and protecting the interests of all stakeholders. For instance, Gull et al. (2023) using samples from 41 countries demonstrates a strong correlation between corporate governance level and CSR practices, that is, the higher the level of board governance, the lower the possibility of corporate social responsibility decoupling [39]. The meaning of governance has evolved from prioritizing shareholders to embracing stakeholder shared governance. This shift necessitates enterprises to fully consider the interests of all pertinent groups and cultivate relationships to establish checks on power and a harmonious balance of interests [40].

On one hand, corporate governance effectively resolves conflicts among stakeholders, eliminating trust crises and creating a better environment for corporate development, thereby gaining competitive advantages. Quality corporate governance reduces operational risks, impacting the risk of stock price collapse. It also influences companies’ hoarding behavior towards bad news by adjusting managers’ mindset [33], supervisory efficiency of corporate executives, and information disclosure [21]. Studies on principle-agency theory argued that corporate governance could be an effective way to address the principal-agent problem [14, 15]. Therefore, effective corporate governance can weaken the positive effect of CSR imbalances on the risk of stock price crashes by reducing possible principal-agent problems of management. Essentially, effective corporate governance serves as a vital tool in building relationships with stakeholders, mitigating risks, and reducing stock price volatility. To some extent, it replaces the function of CSR equilibrium and reduces stock price crash risk.

On the other hand, corporate governance can reduce the risk of future stock price crashes by effectively monitoring and curbing management’s bad news hoarding behavior [41]. In addition, firms with better corporate governance have more analysts following, reducing the degree of surplus management and has a deterrent effect on corporate violations [42]. Similar studies suggest that high analyst attention to firms and institutional investor monitoring moderates to some extent the effect of CSR equilibrium on stock price crashes [43]. Accordingly, corporate governance could play an enhanced role in the mechanism between CSR equilibrium and corporate stock price crash risk. In other words, CSR and corporate governance may play complementary roles in reducing the risk of stock price collapse. Therefore, the effects of corporate governance and CSR equilibrium on the risk of corporate stock price collapse may also have complementary effects that promote each other. Hence, the relationship between CSR equilibrium may be mutually reinforcing, where stronger corporate governance enhances the impact of CSR equilibrium on stock price crash risk. Therefore, we propose the following competing hypotheses.

H2a: There is a substitution effect of corporate governance and social responsibility equilibrium on corporate stock price crash risk

H2b: There is a complementary effect of corporate governance and social responsibility equilibrium on corporate stock price crash risk.

### The heterogeneous effect of nature of ownership on CSR equilibrium and the stock price crash risk

Studies have highlighted that the primary reason private enterprises actively engage in CSR is to enhance legitimacy and access novel resources and social support [44, 45]. In contrast, for state-owned enterprises, CSR is not only a specific, mandatory, and legal corporate goal but also encompasses economic, legal, political, and charitable responsibilities to meet societal expectations [46]. State-owned enterprises face increased scrutiny and expectations from the government, the public, and the media, leading stakeholders to have higher demands for CSR equilibrium [47]. When state-owned enterprises fail to protect the interests of a party due to principal-agent issues, stakeholders tend to be more disillusioned, thereby increasing the risk of share price collapse.

This study argues that maintaining CSR equilibrium reduces the likelihood of stock price collapse by diminishing the probability of managers hiding adverse news. In simpler terms, the more probable it is for managers to conceal bad news, the more beneficial CSR equilibrium becomes in preventing stock price crash. State ownership may lead to conflicting corporate objectives and more intricate principal-agent relationships compared to non-state-owned firms leading to reduced transparency in corporate information [48]. For example, state-owned enterprises have multiple conflicting objectives significantly increasing the agency costs of implementing profit sharing [31, 48]. Also, Managers of state-owned enterprises place a higher value on their political reputation. Due to their proximity to the government, state-owned enterprises may find it easier and less costly to hide negative news. Consequently, the study posits that state-owned firms have a higher probability of concealing adverse news compared to private firms. Therefore, CSR equilibrium is more likely to mitigate the risk of stock price collapse in state-owned firms. Consequently, we propose the following hypothesis.

H3: The effect of social responsibility equilibrium on the risk of stock price collapse is more significant in state-owned firms compared to non-state-owned firms.

## Research design

### Sample and data collection

In order to exam the impact of CSR equilibrium on stock price crash, explore the moderating effect of corporate governance level, and the heterogeneous role of ownership, we selected firms in Shanghai and Shenzhen A-share listed companies (2011–2020) as the research subjects. Since we use the socially responsible equilibrium in one year to explain the risk of a stock price crash in the following year, the sample interval for the explanatory variables is 2010–2019 and the sample interval for the risk of a stock price crash is 2011–2020.

With reference to the existing literature [37], in order to avoid the effect of anomalous samples, we screened the initial samples with the following steps. First, to ensure the reliability of the index of stock price crash risk, we excluded companies with less than 30 annual weekly returns observations. Second, we eliminated company annual observations with data missing. Finally, the sample of ST and *ST companies were further excluded to avoid the influence of data outliers and extreme values. The main variables are winsorized by 1% up and down, resulting in a valid sample size of 13,191. Data of stock price crash risk and corporate governance level, corporate financial and attribute indicators were obtained from the CSMAR database. CSMAR, also known as the China Economic and Financial Research Database, was developed by Shenzhen Sigma Data Technology Co., Ltd. to cater to the needs of academic research. It was created with reference to international renowned databases. CSMAR is tailored to the specifics of the domestic financial market and the research practices of academic institutions and organizations, making it a highly precise database for research purposes. Widely utilized by numerous Chinese universities and research institutes.

Data of CSR were collected through Third-party rating agencies named Hexun. Founded in 1996, Hexun is the developer and builder of the STAQ system, the earliest trading marketplace for securities venues in China. As a third-party rating agency, its CSR rating data on Chinese listed companies is widely used.

### Measures

#### Dependent variable

Stock price crash risk serves as the dependent variable in this study. Existing research mostly uses stock price crash risk indicators to measure the possibility of stock price plummeting [37, 49]. Following previous research, two indicators, NCSKEW (negative conditional return skewness) and DUVOL (down-to-up volatility) were selected to measure the stock price crash risk of the company.

The calculation of the indicators is consistent with the above studies. The higher the value of the resulting indicators, the higher stock price crash risk. We employ l-year-ahead NCSKEW (*NCSKEW*_*t*+1_) and DUVOL (*DUVOL*_*t*+1_) as the dependent variables in our empirical tests below.

#### Explanatory variable

CSR equilibrium serves as the explanatory variable in this study. Previous studies adopted various methods such as content analysis, reputation, and CSR accounting to design index system to measure CSR situation [8, 50]. However, CSR evaluation by individuals is often subjective. With reference to the ideas of existing literature, we select a third-party rating agency Hexun with more objectivity and authority, to collect various scores of CSR to measure CSR fulfillment [50, 51]. Hexun measures the social responsibility performed by enterprises in five dimensions: shareholder responsibility, employee responsibility, supplier responsibility, environmental responsibility, and charity responsibility. We draw on the principle of variance to design the equilibrium degree indicator VARCSR, which is modeled as follows:

$$VARCSR=\frac{1}{N}\sum_{i=1}^{N}\;{\left({CSR}_{i}-AVECSR\right)}^{2}$$ (1)

*CSR*_*i*_ denotes shareholder responsibility, employee responsibility, supplier responsibility, environmental responsibility, and charity responsibility; AVECSR is the mean value of social responsibility in the five dimensions. The smaller the VARCSR, the more equilibrious the CSR fulfillment is. In addition, in the robustness test, the reliability of the regression results is confirmed by normalizing the social responsibility of different dimensions and then further measuring the social responsibility equilibrium degree, referring to the method of Tewari & Bhattacharya (2023) [50].

#### Moderate variable

Corporate governance level serves as the moderate variable in this study. Based on established research [11, 52], this study applied principal component analysis and selected 4 indicators to construct a corporate governance index. The 4 indicators include the supervision of the board of directors (i.e., the proportion of independent directors and the size of the board of directors), the supervision of the shareholding structure (i.e., the proportion of institutional shareholdings and the degree of checks and balances on shareholdings), the incentives for executives (i.e., the remuneration of executives and the proportion of shareholdings of executives), and the decision-making power of the general manager (i.e., whether the chairman of the board and the general manager are combined in one position).

### Control variables

Control variables need to be closely related to stock price crash risk in order to alleviate the problem of omitted variables. Integrating relevant theories and literature studies [53, 54], we selected the average monthly share turnover (Dturn), standard deviation of firm-specific weekly return (Sigma), the mean of firm-specific weekly returns (Ret), firm’s total assets (Size), the book-to-market ratio (BM), debt asset ratio (Lev), return on equity (ROE) and information transparency (DD) as control variables. In addition, we control for year and industry fixed effects. Table 1 summarizes the definitions of the variables.

**Table 1:** Definition of main indicators.

Variables	Symbols	Variable Definition	References
Dependent variable	*NCSKEW*_*t*+1_	Negative conditional return skewness in period t+1	Kim et al. (2011) Xu et al., (2014)
	*DUVOL*_*t*+1_	The ratio of down-to-up volatility in earnings in period t+1	
Explanatory variable	*VARCSR*	The specific calculation method is referred to Formula (1). The greater the VARCSR, the lower the degree of social responsibility equilibrium	Fatima et al. (2023) Tewari & Bhattacyharya (2023)
Moderating variable	*GOV*	Corporate governance level, obtained by first principal component analysis	Oh et al. (2018) Chan et al (2014)
Control variables	*Size*	The log of the firm’s total assets	Hutton et al. (2009) Wang & Qian (2011)
	*Lev*	total long-term debts divided by total assets	
	*ROE*	Rate of return on common stockholders’ equity, net income divided by shareholders’ equity.	
	*BM*	Book-to-market ratio, book value divided by total market value	
	*Dturn*	The average monthly share turnover over the current fiscal year period minus the average monthly share turnover over the previous fiscal year period, where monthly share turnover is calculated as the monthly trading volume divided by the total number of shares outstanding during the month	
	*DD*	Information transparency, measured by the absolute value of manipulable accrued profits	
	*Ret*	The mean of firm-specific weekly returns over the fiscal year period, times 100	
	*Sigma*	the standard deviation of firm-specific weekly returns over the fiscal year period	

NCSKEW_t+1_, Negative conditional return skewness in period t+1; DUVOL_t+1_, The ratio of down-to-up volatility in earnings in period t+1; VARCSR, degree of corporate social responsibility equilibrium; GOV, Corporate governance level; Lev, debt asset ratio; ROE, return on equity; BM, Book-to-market ratio; Dturn, monthly share turnover; DD, information transparency; Ret, the mean of firm-specific weekly returns over the fiscal year period; Sigma, standard deviation of firm-specific weekly return

### Model specification

To verify the impact of social responsibility equilibrium on stock price crash risk and the moderating effect of the corporate governance level, the following regression model is constructed.


$${Cras\hbar }_{i,t+1}=\alpha_{0}+\alpha_{1}{VARCSR}_{it}+{\beta Z}_{it}+u_{i}+\lambda_{t}+v_{it}$$ (2)

$${Cras\hbar }_{i,t+1}=\alpha_{0}+\alpha_{1}{VARCSR}_{it}+\alpha_{2}{GOV}_{it}+\alpha_{3}{VARCSR}_{it}*{GOV}_{it}+{\beta Z}_{it}+u_{i}+\lambda_{t}+v_{it}$$ (3)

VARCSR is used in the model to measure the degree of social responsibility equilibrium. *Crasℏ*_*i*,*t*+1_ are two indicators of stock price crash risk NCSKEW and DUVOL in period t+1. GOV is an indicator of corporate governance level. To confirm the moderating effect of corporate governance, we introduce the interaction term GOV*VARCSR between social responsibility equilibrium and corporate governance. In addition, Z are control variables. *u*_*i*_, *λ*_*t*_ and *v*_*it*_ are the individual effects that do not vary with time, the time effects that do not vary with individuals, and the random error terms that vary both with time and with individuals, respectively.

## Empirical test results

### Descriptive statistics and correlation analysis

Descriptive statistics are presented in Table 2. The mean *NCSKEW*_*t*+1_ is -0.373 and the standard deviation is 0.632. The mean *DUVOL*_*t*+1_ is -0.251 and the standard deviation is 0.431. The results indicate differences in the risk of stock price crash among the sample companies. The mean value of VARCSR is 31.47 and the standard deviation is 20.27, indicating a large difference in social responsibility equilibrium among different listed companies. In addition, the minimum value of VARCSR is only 0.00750, indicating that some companies have almost the same degree of fulfillment for each dimension of social responsibility, leading to a very equilibrious social responsibility. The mean value of GOV is-0.303, indicating that the governance level of Chinese listed companies is still relatively low, with the minimum value of-1.281 and the maximum value of 1.641.

**Table 2:** Descriptive statistics of main variables (2010–2019).

Variables	(1) N	(2) Average value	(3) Standard deviation	(4) Minimum value	(5) Maximum value
*NCSKEW*_*t*+1_	13,191	-0.373	0.632	-1.671	0.754
*DUVOL*_*t*+1_	13,191	-0.251	0.431	-1.034	0.545
*VARCSR*	13,191	31.47	20.27	0.00750	139.6
*GOV*	13,191	-0.303	0.782	-1.281	1.641
*Size*	13,191	22.53	1.311	17.64	28.64
*Lev*	13,191	0.476	0.200	0.00708	1.056
*ROE*	13,191	0.0635	0.171	-6.850	1.751
*BM*	13,191	1.292	1.395	0.00986	19.15
*Dturn*	13,191	-0.0403	0.318	-2.479	1.917
*DD*	13,191	-0.0367	0.0281	-0.297	-0.000649
*NCSKEW*	13,191	-0.322	0.726	-0.271	2.139
*DUVOL*	13,191	-0.216	0.478	-1.588	1.272
*Ret*	13,191	0.00150	0.008870	-0.2193	0.0650
*Sigma*	13,191	0.05948	0.02327	0.01933	0.226

NCSKEW_t+1_, Negative conditional return skewness in period t+1; DUVOL_t+1_, The ratio of down-to-up volatility in earnings in period t+1; VARCSR, degree of corporate social responsibility equilibrium; GOV, Corporate governance level; Lev, debt asset ratio; ROE, return on equity; BM, Book-to-market ratio; Dturn, monthly share turnover; DD, information transparency; Ret, the mean of firm-specific weekly returns over the fiscal year period; Sigma, standard deviation of firm-specific weekly return; N, number.

To test whether the multicollinearity problem among variables affects the relationship between CSR equilibrium and stock price crash risk, we first make a preliminary judgment on the variables in the model using Pearson correlation analysis. Table 3 shows that most of the variables put into the same model for regression have correlation coefficients below 0.5, indicating that the relationship between the variables is not disturbed by multicollinearity problems. The correlation coefficients of *NCSKEW*_*t*+1_ and *DUVOL*_*t*+1_ have significant correlation coefficients at the level of p<0.01 and are as high as 0.877, proving that these two measures of stock price crash risk have a high degree of consistency. The social responsibility equilibrium measure, VARCSR, has a positive effect on stock price crash risk at the level of p<0.01, tentatively confirming that the lower the social responsibility equilibrium, the higher stock price crash risk of the company. However, the correlation analysis does not consider the influence of other factors on stock price crash risk, so we further test the relationship between social responsibility equilibrium and stock price crash risk through multiple regression analysis.

**Table 3:** PEARSON correlation test.

	1	2	3	4	5	6	7	8	9	10	11	12	13
*NCSKEW*_*t*+1_	1												
*DUVOL*_*t*+1_	0.877***	1											
*VARCSR*	0.047***	0.038***	1										
*Size*	-0.034***	-0.053***	0.226***	1									
*Lev*	-0.034***	-0.040***	-0.181***	0.433***	1								
*ROE*	0.051***	0.047***	0.416***	0.128***	-0.117***	1							
*BM*	-0.108***	-0.106***	-0.007	0.608***	0.536***	-0.047***	1						
*Dturn*	-0.068***	-0.083***	0.002	0.019***	0.011	-0.067***	-0.018**	1					
*DD*	0.00400	-0.001	0.148***	0.238***	0.047***	0.179***	0.170***	-0.040***	1				
*NCSKEW*	0.062***	0.054***	0.020**	-0.053***	-0.041***	-0.010	-0.044***	-0.076***	-0.025***	1			
*DUVOL*	0.052***	0.048***	0.0120	-0.075***	-0.053***	-0.014	-0.040***	-0.082***	-0.022**	0.879***	1		
*Ret*	0.053***	0.037***	0.068***	-0.032***	-0.004***	0.105***	-0.180**	0.469***	0.015*	-0.182***	-0.198***	1	
*Sigma*	-0.038***	-0.041***	-0.131***	-0.205***	-0.003***	-0.100***	-0.219***	0.468***	-0.128***	-0.127***	-0.127***	0.508***	1

NCSKEW_t+1_, Negative conditional return skewness in period t+1; DUVOL_t+1_, The ratio of down-to-up volatility in earnings in period t+1; VARCSR, degree of corporate social responsibility equilibrium; GOV, Corporate governance level; Lev, debt asset ratio; ROE, return on equity; BM, Book-to-market ratio; Dturn, monthly share turnover; DD, information transparency; Ret, the mean of firm-specific weekly returns over the fiscal year period; Sigma, standard deviation of firm-specific weekly return

*, **, *** indicate significant at 10%, 5%, 1% confidence level, respectively

### Full-sample regression

The results of the regressions are presented in Table 4. Columns 1–2 validate the relationship between social responsibility equilibrium and stock price crash risk using *NCSKEW*_*t*+1_ and *DUVOL*_*t*+1_ as stock price crash risk measures, respectively. Columns 3–4 examine the moderating effect of corporate governance on the relationship between social responsibility equilibrium and stock price crash risk after adding the interaction term between corporate governance level and social responsibility equilibrium. From columns 1–2 of Table 4, it is found that the inverse indicator of social responsibility equilibrium, VARCSR, has a positive effect on stock price crash risk (*NCSKEW*_*t*+1_ and *DUVOL*_*t*+1_) at the level of p<0.01 and p<0.05, respectively. Since VARCSR are an inverse measure of social responsibility equilibrium variable, this shows that social responsibility equilibrium significantly reduces stock price crash risk, further confirming hypothesis 1. This indicates CSR equilibrium helps prevent managers from viewing social responsibility solely as a ’self-interest tool’, as noted in studies by scholars like Hsueh & Chang (2008) and Bian et al (2021) on the link between social responsibility and stock price crash risk. Maintaining this equilibrium is crucial for enhancing the stability of a company’s stock price.

**Table 4:** The results of the full-sample regression.

	(1) *NCSKEW*_*t*+1_	(2) *DUVOL*_*t*+1_	(3) *NCSKEW*_*t*+1_	(4) *DUVOL*_*t*+1_
*VARCSR*	0.001***	0.001**	0.001**	0.000*
	(0.000)	(0.000)	(0.000)	(0.000)
VARCSR*Gov			-0.001***	-0.001*
			(0.000)	(0.000)
*Size*	0.009	-0.005	0.012*	-0.003
	(0.006)	(0.004)	(0.006)	(0.004)
*Lev*	0.072**	0.047*	0.080**	0.053**
	(0.036)	(0.025)	(0.036)	(0.025)
*ROE*	0.003	0.011	0.010	0.015
	(0.036)	(0.025)	(0.036)	(0.025)
*BM*	-0.037***	-0.019***	-0.037***	-0.019***
	(0.006)	(0.004)	(0.006)	(0.004)
*Dturn*	-0.013	-0.011	-0.014	-0.011
	(0.024)	(0.016)	(0.024)	(0.016)
*DD*	-0.094	-0.081	-0.028	-0.038
	(0.206)	(0.140)	(0.206)	(0.141)
*NCSKEW*	0.062***		0.061***	
	(0.008)		(0.008)	
*DUVOL*		0.052***		0.051***
		(0.008)		(0.008)
*Ret*	11.848***	8.018***	11.990***	8.110***
	(0.988)	(0.677)	(0.988)	(0.677)
*Sigma*	-0.952**	-0.885***	-1.056***	-0.956***
	(0.408)	(0.277)	(0.409)	(0.278)
*Gov*			0.050***	0.031***
			(0.013)	(0.009)
*_cons*	-0.548***	-0.110	-0.583***	-0.136
	(0.136)	(0.093)	(0.137)	(0.094)
r2_a	0.061	0.059	0.062	0.060
F	31.757	26.623	27.768	23.323
N	13191	13191	13191	13191

NCSKEW_t+1_, Negative conditional return skewness in period t+1; DUVOL_t+1_, The ratio of down-to-up volatility in earnings in period t+1; VARCSR, degree of corporate social responsibility equilibrium; GOV, Corporate governance level; Lev, debt asset ratio; ROE, return on equity; BM, Book-to-market ratio; Dturn, monthly share turnover; DD, information transparency; Ret, the mean of firm-specific weekly returns over the fiscal year period; Sigma, standard deviation of firm-specific weekly return; cons, constant; r2-a, Adjusted R-squared; F, variance; N, number

*, **, *** represent significant at 10%, 5% and 1% levels, respectively; Robust standard error in parentheses.

From columns 3–4 of Table 4, We find that whether using *NCSKEW*_*t*+1_ or *DUVOL*_*t*+1_ as an index of stock price crash risk, the corporate governance level has a significant impact on the relationship between social responsibility equilibrium and stock price crash risk at the level of p<0.01 and p<0.05. The regression coefficient of the interaction term is negative, indicating that corporate governance level weakens the negative influence of social responsibility equilibrium on stock price crash risk, and the relationship between social responsibility equilibrium and corporate governance is substitution. Hence, hypothesis 2a is verified.

### Endogeneity checks

In this study, we utilized lagged one-period stock price crash risk as the dependent variable to mitigate the endogeneity issue stemming from the reverse causality between CSR equilibrium and stock price crash risk. Nevertheless, acknowledging the potential presence of omitted variables influencing corporate stock price crash risk in the model, we also employed the instrumental variable method to further address endogeneity concerns.

Specifically, the existing literature mainly uses the mean value of social responsibility report scores in the same year and the same region as an instrumental variable of CSR [55]. The mean value represents the average level of CSR, and the social responsibility performed by the enterprises in the same year and in the same region will be influenced by other enterprises due to the competition and the environment they are in. We argue that social responsibility equilibrium also satisfies the above conditions. Other enterprises will inevitably influence social responsibility equilibrium of a single enterprise, and the mean of social responsibility equilibrium is less likely to affect the crash risk of individual stocks, which meets the correlation and exogeneity requirements of instrumental variables. The final results are shown in Table 5. From the regression results, it can be found that the effect of instrumental variables of CSR equilibrium on stock crash risk is consistent with the full-sample regression.

**Table 5:** Endogeneity test: Regression results for instrumental variables.

	(1) *NCSKEW*_*t*+1_	(2) *DUVOL*_*t*+1_	(3) *NCSKEW*_*t*+1_	(4) *DUVOL*_*t*+1_
*VARCSR (IV)*	0.009*	0.002	0.007*	0.000
	(0.005)	(0.003)	(0.004)	(0.003)
VARCSR*Gov(IV)			-0.002	-0.002*
			(0.002)	(0.001)
*Size*	-0.026	-0.011		
	(0.022)	(0.015)		
*Lev*	0.255**	0.077	0.183**	0.030
	(0.116)	(0.077)	(0.082)	(0.055)
*ROE*	-0.304	-0.038	-0.274	0.007
	(0.187)	(0.124)	(0.193)	(0.130)
*BM*	-0.022**	-0.017**	-0.032***	-0.020***
	(0.011)	(0.007)	(0.005)	(0.003)
*Dturn*	-0.028	-0.013	-0.053**	-0.027*
	(0.026)	(0.017)	(0.022)	(0.015)
*DD*	-0.248	-0.105		
	(0.230)	(0.153)		
*NCSKEW*	0.056***		0.066***	
	(0.009)		(0.007)	
*DUVOL*		0.051***		0.059***
		(0.009)		(0.008)
*Ret*	8.485***	7.474***	9.667***	8.086***
	(2.252)	(1.511)	(1.928)	(1.303)
*Sigma*	0.597	-0.636	0.850	-0.614
	(1.017)	(0.678)	(1.077)	(0.729)
*Gov*			0.101	0.102**
			(0.073)	(0.049)
r2_a	-0.028	0.014	-0.008	0.016
F	29.773	25.967	43.941	38.698
N	13191	13191	13191	13191

NCSKEW_t+1_, Negative conditional return skewness in period t+1; DUVOL_t+1_, The ratio of down-to-up volatility in earnings in period t+1; VARCSR, degree of corporate social responsibility equilibrium; GOV, Corporate governance level; Lev, debt asset ratio; ROE, return on equity; BM, Book-to-market ratio; Dturn, monthly share turnover; DD, information transparency; Ret, the mean of firm-specific weekly returns over the fiscal year period; Sigma, standard deviation of firm-specific weekly return; cons, constant; r2-a, Adjusted R-squared; F, variance; N, number

*, **, *** represent significant at 10%, 5% and 1% levels, respectively; Robust standard error in parentheses.

### Robustness tests

The index data used to measure CSR equilibrium in this study primarily originates from the third-party rating agency Hexun, which gathers diverse CSR scores. While these scores do provide insight into the extent of CSR fulfillment across various dimensions, there are quantitative variations within each dimension. This variability could potentially skew the overall ’equilibrium’ if certain dimensions excel, leading to misinterpretation. Thus, this study conducts a robustness test that standardizes the different dimensions of CSR prior to assessing CSR equilibrium. Table 6 shows the results after normalizing the data. From columns 1 and 2, it is found that the VARCSR coefficient is positive, i.e., the social responsibility equilibrium reduces stock price crash risk. From columns 3 and 4, it is found that the VARCSR2*Gov coefficient is negative, i.e., corporate governance level weakens the negative effect of social responsibility equilibrium on stock price crash risk. Therefore, the results are robust.

**Table 6:** The results of robustness checks.

	(1) *NCSKEW*_*t*+1_	(2) *DUVOL*_*t*+1_	(3) *NCSKEW*_*t*+1_	(4) *DUVOL*_*t*+1_
*VARCSR*	0.511**	0.336**	0.323	0.245*
	(0.243)	(0.166)	(0.229)	(0.145)
VARCSR*Gov			-0.618***	-0.313**
			(0.223)	(0.152)
*Size*	0.013**	-0.003	0.012**	-0.004
	(0.006)	(0.004)	(0.006)	(0.004)
*Lev*	0.061*	0.042*	0.032	0.024
	(0.036)	(0.024)	(0.032)	(0.022)
*ROE*	0.011	0.014	0.024	
	(0.036)	(0.025)	(0.035)	
*BM*	-0.039***	-0.020***	-0.037***	-0.019***
	(0.006)	(0.004)	(0.005)	(0.004)
*Dturn*	-0.012	-0.010	-0.037*	
	(0.024)	(0.016)	(0.019)	
*DD*	-0.107	-0.091		
	(0.206)	(0.141)		
*NCSKEW*	0.062***		0.070***	
	(0.008)		(0.007)	
*Ret*	11.960***	8.061***	12.402***	8.216***
	(0.988)	(0.677)	(0.861)	(0.584)
*Sigma*	-1.019**	-0.914***	-0.706*	-0.897***
	(0.407)	(0.277)	(0.361)	(0.236)
*DUVOL*		0.052***		0.060***
		(0.008)		(0.007)
*Gov*			0.068***	0.040***
			(0.016)	(0.011)
*_cons*	-0.617***	-0.152	-0.568***	-0.105
	(0.136)	(0.093)	(0.126)	(0.084)
r2_a	0.061	0.059	0.065	0.062
F	31.316	26.412	42.462	43.568
N	13191	13191	13191	13191

NCSKEW_t+1_, Negative conditional return skewness in period t+1; DUVOL_t+1_, The ratio of down-to-up volatility in earnings in period t+1; VARCSR, degree of corporate social responsibility equilibrium; GOV, Corporate governance level; Lev, debt asset ratio; ROE, return on equity; BM, Book-to-market ratio; Dturn, monthly share turnover; DD, information transparency; Ret, the mean of firm-specific weekly returns over the fiscal year period; Sigma, standard deviation of firm-specific weekly return; cons, constant; r2-a, Adjusted R-squared; F, variance; N, number

*, **, *** represent significant at 10%, 5% and 1% levels, respectively; Robust standard error in parentheses.

### Heterogeneity test: Enterprise nature

While the above results have demonstrated that achieving CSR equilibrium can reduce stock price crash risk. Additionally corporate governance can partially act as a substitute role for social responsibility equilibrium, i.e., weaken the negative impact of social responsibility equilibrium on stock price crash risk. However, considering that enterprises of different ownership natures have different resources and motivation to fulfill social responsibility may also lead to different effects of social responsibility equilibrium fulfillment on stock price crash risk, we further explore the differences in the relationship between social responsibility equilibrium and stock price crash risk for firms with different ownership natures by dividing firms into state-owned and non-state-owned.

Table 7 shows the regression results for different ownership natures. Columns 1 and 3 are the regression results for state-owned enterprises, and columns 2 and 4 are the regression results for non-state-owned enterprises. The results show that for state-owned enterprises, social responsibility equilibrium can significantly reduce stock price crash risk, but the relationship between social responsibility equilibrium and stock price crash for non-state-owned enterprises is not significant. Compared with non-state-owned enterprises, state-owned enterprises need to fulfill their social responsibility in a equilibrious manner to meet the expectations of all stakeholders and reduce the risks and costs caused by principal-agent problems. Therefore, the negative effect of social responsibility equilibrium on stock price crash risk is more significant in state-owned enterprises, which further validates the main logic of this study.

**Table 7:** The result of the regression test from the aspect of ownership structure.

	(1) State-owned *NCSKEW*_*t*+1_	(2) Non-State *NCSKEW*_*t*+1_	(3) State-owned *DUVOL*_*t*+1_	(4) Non-State *DUVOL*_*t*+1_
*VARCSR*	0.002***	0.000	0.001***	0.000
	(0.000)	(0.000)	(0.000)	(0.000)
*Size*	0.001	0.024**	-0.008	0.001
	(0.008)	(0.009)	(0.006)	(0.006)
*Lev*	0.059	0.093*	0.041	0.061*
	(0.053)	(0.051)	(0.036)	(0.034)
*ROE*	0.035	-0.012	0.029	0.001
	(0.054)	(0.049)	(0.037)	(0.033)
*BM*	-0.024***	-0.055***	-0.012**	-0.028***
	(0.007)	(0.010)	(0.005)	(0.007)
*Dturn*	-0.010	-0.009	-0.021	-0.000
	(0.037)	(0.031)	(0.025)	(0.021)
*DD*	0.431	-0.306	0.259	-0.212
	(0.337)	(0.266)	(0.231)	(0.181)
*NCSKEW*	0.066***	0.051***		
	(0.011)	(0.011)		
*Ret*	13.111***	11.482***	9.085***	7.507***
	(1.565)	(1.323)	(1.077)	(0.902)
*Sigma*	-1.181*	-0.973*	-1.129***	-0.783**
	(0.633)	(0.545)	(0.430)	(0.369)
*DUVOL*			0.052***	0.045***
			(0.012)	(0.011)
*_cons*	-0.381**	-0.819***	-0.052	-0.217
	(0.189)	(0.206)	(0.130)	(0.140)
r2_a	0.062	0.059	0.056	0.061
F	17.550	15.130	13.884	12.260
N	6554	6637	6554	6637

NCSKEW_t+1_, Negative conditional return skewness in period t+1; DUVOL_t+1_, The ratio of down-to-up volatility in earnings in period t+1; VARCSR, degree of corporate social responsibility equilibrium; GOV, Corporate governance level; Lev, debt asset ratio; ROE, return on equity; BM, Book-to-market ratio; Dturn, monthly share turnover; DD, information transparency; Ret, the mean of firm-specific weekly returns over the fiscal year period; Sigma, standard deviation of firm-specific weekly return; cons, constant; r2-a, Adjusted R-squared; F, variance; N, number

*, **, *** represent significant at 10%, 5% and 1% levels, respectively; Robust standard error in parentheses.

## Discussion

The main purpose of this study is to exam the effects CSR equilibrium have on stock price crash risk. In line with previous studies [5, 56], we argue that CSR fulfillment could have a positive role on reducing stock price crash risks. This study enriches the existing research by exploring CSR from a balanced perspective instead of focusing on a certain type, and providing an effective supplement to how to fulfill CSR. Existing research divides CSR into different dimensions [51], and the research conclusions on the impact of companies’ fulfillment of different types of social responsibilities on their stock prices are not consistent [57]. Result of this study demonstrates that the more balanced the fulfillment of CSR, the lower the risk of its estimated collapse. Unlike studies implied that certain type of CSR fulfillment could eliminate stock price crash risks, this research emphasizes the importance of equilibrium of CSR. According to the result, the more a company implements all aspects of social responsibility in a balanced manner, the higher its information transparency, the lower the possibility of principal-agent problems, and the smaller the risk of stock price collapse.

Moreover, statistic results demonstrate that the role of CSR equilibrium is greater for state-owned enterprises compared to private enterprises. It is understandable that structure of ownership could influence quality of CSR reporting [58], and diverse groups of shareholders have different impacts on the firm’s CSR involvement [59]. When government serves as the controlling shareholder of the company, it may be more incentive to pursue social and environmental stability [60]. Therefore, on the one hand, state-owned enterprises will devote more resources to fulfill, or even over-fulfill, their social responsibilities. Also, the number of shares held by governments in companies will give them the power to intervene in such entities to report additional information in order to satisfy public expectation [61]. Similar behavior raises agency costs, which in turn increases the risk of a firm’s estimated collapse. Therefore, it is more important to have a balanced approach to CSR in state-own companies rather than focusing on one certain type of CSR.

Meanwhile, there is a substitution between corporate governance capacity and CSR equilibrium in terms of its effect on stock price collapse. This finding echoes the theoretical line that corporate governance serves as a foundation for CSR [1, 62]. More importantly this finding deepens the existing research by focusing on the interdependence between corporate governance and CSR. Corporate governance could be interpreted as rights among all parties with a stake in the company. The more excellent a firm’s corporate governance capability is, the clearer the rights and responsibilities between the firm’s various stakeholders are, and the fewer principal-agent problems there are [63, 64]. When agency costs are reduced, the more likely and motivated an enterprise is to fulfill its social responsibilities in a balanced manner. This could further lead to productive business-related benefits [42, 59], and therefore lower the stock price crash risks.

The main theoretical contributions are two folds. Firstly, this study contributes to the existing research on CSR through the lens of principle-agent theory. Within the framework of principle-agent theory, corporate social responsibility disclosure is categorized into quantity and quality of information disclosure [18, 42]. While previous studies have primarily examined the quantity of corporate social responsibility fulfillment, noting conflicting effects on estimated collapse risk [16]. This study delves into the quality of such fulfillment by analyzing CSR equilibrium. CSR equilibrium refers to the dynamic balance of corporate social responsibility fulfillment in different dimensions. On this basis, there is a lack of motivation among different stakeholders to break this balance, thus maintaining the stable development of the enterprise. This research conclusion not only responds to research examining CSR from a dynamic perspective [8, 23], but also enriches researchers’ understanding of the effectiveness of CSR implementation.

Secondly, findings enrich researchers’ understanding of how CSR helps reduce firms’ stock price crush risks. Consistent with previous research [38, 43], the empirical findings of this study demonstrate that CSR equilibrium can enhance information transparency, decrease agency costs, and consequently mitigate the likelihood of stock price crashes. Furthermore, this study innovatively combines CSR and corporate governance theories to reveal that the impact of balanced CSR fulfillment and effective corporate governance on stock price volatility varies. This finding expands the strategic potential of corporate social responsibility. The study found that this substitution effect is more obvious in state-owned enterprises, which further illustrates that the balanced fulfillment of social responsibilities by enterprises in different institutional environments may have multiple effects such as attracting innovative resources and gaining social support. This finding expands the strategic potential of corporate social responsibility [65]. The study found that this substitution effect is more obvious in state-owned enterprises, which further illustrates that the balanced fulfillment of social responsibilities by enterprises in different institutional environments may have multiple effects such as attracting innovative resources and gaining social support.

## Conclusion and future lines

The primary findings of the study indicate that achieving CSR equilibrium could significantly reduce the risk of future stock price crashes. While the level of CSR fulfillment may not directly predict the risk of stock price collapse, a well-balanced implementation of CSR can often reduce the probability of using CSR as a "whitewash tool" and effectively reduce the likelihood of stock price collapse caused by sudden and vicious events. Furthermore, there exists a notable substitution effect between CSR equilibrium and corporate governance concerning firms’ share price crash risk. The level of corporate governance weakens the negative effect of the CSR equilibrium on the risk of stock price collapse, i.e., the effect of the social responsibility equilibrium on the risk of stock price collapse is more pronounced in firms with a weak level of governance. Additionally, the study reveals that the impact of CSR equilibrium on the stock price collapse risk varies depending on ownership structure of firms. State-owned companies, which are more likely to conceal negative information, experience a more pronounced effect of CSR equilibrium in reducing the risk of stock price collapse compared to private firms. This finding further underscores the notion that achieving CSR equilibrium can help lower the risk of a firm’s stock price collapse by inhibiting management’s behavior of hiding bad news, increasing information transparency, and reducing agency costs.

This research provides valuable managerial insights. First, enterprises need to understand that fulfilling CSR goes beyond seeking short-term financial advantages, it involves integrating the enterprise into social development trend and achieving ethical management [66]. If CSR is selectively used by self-interested management, it will bring impactful negative consequences to the company. Balanced social responsibility can effectively reduce the probability of negative publicity. Second, social responsibility rating agencies should not solely focus the level of CSR investment. It is crucial for authoritative evaluation agencies to incorporate CSR equilibrium into the basis of social responsibility ratings, review the authenticity and efficacy of CSR performance reports, and highlight potential liability risks in CSR reporting. Third, when investors evaluate a company’s CSR performance, they cannot only consider a certain dimension of the company’s responsibility information [36]. It would be more meaningful for them to assess the balance of CSR efforts and discern companies’ genuine commitment to it.

In order to further enrich CSR equilibrium research, future researchers can focus on the following research topics. First, this paper measures the CSR equilibrium using data from listed companies, which expands the existing knowledge on CSR. However, the information disclosed in annual reports and CSR reports of listed companies is limited. Therefore, subsequent studies can adopt methods such as case and group analysis to further study CSR equilibrium and deepen the theoretical explanatory ability. Second, we argue that there is a linear relationship between CSR equilibrium and stock price crash risks from principal-agent theory by focusing on the quality of CSR practice. Studies drawing on quantity of CSR disclosure explores the inverted u-shaped relationship between the number of corporate social responsibility information disclosures and stock price crash [67]. Research on CSR equilibrium just started, and different theoretical relationships between it and stock price crash risk could be hypothesized based on different theories. Therefore, whether there is a nonlinear relationship between CSR equilibrium and corporate stock price crash may become another research direction in related fields in the future.

## Supporting information

S1 Dataset Additional company data are available on CSMAR (https://data.csmar.com). The relevant data of CSR equilibrium can be download conditionally on Hexun (https://data.hexun.com).
(XLSX)

## References

[1] Filatotchev I, Stahl GK. Towards transnational CSR. Corporate social responsibility approaches and governance solutions for multinational corporations. Organ Dyn. 2015 Feb 24;44(2):121–9.

[2] Huang X ‘“Beryl”‘, Watson L. Corporate social responsibility research in accounting. J Account Lit. 2015 Jan 1;34(1):1–16.

[3] Thuy CTM, Trung TQ, Khuong NV, Liem NT. From Corporate Social Responsibility to Stock Price Crash Risk: Modelling the Mediating Role of Firm Performance in an Emerging Market. Sustainability. 2021 Jan;13(22):12557.

[4] Huang W, Chen S, Nguyen LT. Corporate Social Responsibility and Organizational Resilience to COVID-19 Crisis: An Empirical Study of Chinese Firms. Sustainability. 2020 Jan;12(21):8970.

[5] Kim Y, Li H, Li S. Corporate social responsibility and stock price crash risk. J Bank Finance. 2014 Jun 1;43:1–13.

[6] Kim J, Ju J, Kim J. The effects of consideration of future consequences and CSR fit in stigmatized industries: Perceived CSR motives as mediators. Public Relat Rev. 2023 Mar 1;49(1):102294.

[7] Ma JZ, Huang HY, Zhu Q, Shen X. Corporate Social Responsibility Disclosure, Market Supervision, and Green Investment. Emerg Mark Finance Trade. 2022 Dec 8;58(15):4389–98.

[8] Bian J, Liao Y, Wang YY, Tao F. Analysis of firm CSR strategies. Eur J Oper Res. 2021 May 1;290(3):914–26.

[9] Dang VC, Nguyen QK. Internal corporate governance and stock price crash risk: evidence from Vietnam. J Sustain Finance Invest. 2024 Jan 2;14(1):24–41.

[10] Wang Q, Li X, Liu Q. Empirical research of accounting conservatism, corporate governance and stock price collapse risk based on panel data model. Connect Sci. 2021 Oct 2;33(4):995–1010.

[11] Oh WY, Chang YK, Kim TY. Complementary or Substitutive Effects? Corporate Governance Mechanisms and Corporate Social Responsibility. J Manag. 2018 Sep 1;44(7):2716–39.

[12] Devinney TM. Is the Socially Responsible Corporation a Myth? The Good, the Bad, and the Ugly of Corporate Social Responsibility. Acad Manag Perspect. 2009 May;23(2):44–56.

[13] Carroll AB. The Four Faces of Corporate Citizenship. Bus Soc Rev. 1998 Sep;100–101(1):1–7.

[14] Carroll AB. A Three-Dimensional Conceptual Model of Corporate Performance. Acad Manage Rev. 1979 Oct;4(4):497–505.

[15] McDaniel RR. Management Strategies for Complex Adaptive Systems Sensemaking, Learning, and Improvisation. Perform Improv Q. 2008 Oct 22;20(2):21–41.

[16] Fatima T, Elbanna S. Corporate Social Responsibility (CSR) Implementation: A Review and a Research Agenda Towards an Integrative Framework. J Bus Ethics. 2023 Feb;183(1):105–21. doi: 10.1007/s10551-022-05047-8 35125567 PMC8807959

[17] Goerzen A, Sartor M, Brandl K, Fitzsimmons S. Widening the lens: Multilevel drivers of firm corporate social performance. J Int Bus Stud. 2023 Feb 1;54(1):42–60.

[18] Velte P. Meta-analyses on Corporate Social Responsibility (CSR): a literature review. Manag Rev Q. 2022 Sep;72(3):627–75.

[19] Blanco-González A, Cachón-Rodríguez G, Hernández-Perlines F, Prado-Román C. Effects of social responsibility on legitimacy and revisit intention: The moderating role of anxiety. J Bus Res. 2023 Mar;157:113583.

[20] Czinkota M, Kaufmann HR, Basile G. The relationship between legitimacy, reputation, sustainability and branding for companies and their supply chains. Ind Mark Manag. 2014 Jan 1;43(1):91–101.

[21] Gatignon A. The double-edged sword of boundary-spanning Corporate Social Responsibility programs. Strateg Manag J. 2022;43(10):2156–84.

[22] Clarkson ME. A Stakeholder Framework for Analyzing and Evaluating Corporate Social Performance. Acad Manage Rev. 1995 Jan;20(1):92–117.

[23] Hsueh CF, Chang MS. Equilibrium analysis and corporate social responsibility for supply chain integration. Eur J Oper Res. 2008 Oct 1;190(1):116–29.

[24] Petrovits CM. Corporate-sponsored foundations and earnings management. J Account Econ. 2006 Sep 1;41(3):335–62.

[25] Prior D, Surroca J, Tribó JA. Are Socially Responsible Managers Really Ethical? Exploring the Relationship Between Earnings Management and Corporate Social Responsibility. Corp Gov Int Rev. 2008 May;16(3):160–77.

[26] Gomes AR. Going Public with Asymmetric Information, Agency Costs and Dynamic Trading [Internet]. Rochester, NY; 1997 [cited 2024 May 12]. Available from: https://papers.ssrn.com/abstract=36566

[27] Verrecchia RE. Essays on disclosure. J Account Econ. 2001 Dec 1;32(1):97–180.

[28] Leland HE. Agency Costs, Risk Management, and Capital Structure. J Finance. 1998;53(4):1213–43.

[29] Kothari SP, Shu S, Wysocki PD. Do Managers Withhold Bad News? J Account Res. 2009;47(1):241–76.

[30] Hemingway CA, Maclagan PW. Managers’ Personal Values as Drivers of Corporate Social Responsibility. J Bus Ethics. 2004 Mar 1;50(1):33–44.

[31] Lin KJ, Tan J, Zhao L, Karim K. In the name of charity: Political connections and strategic corporate social responsibility in a transition economy. J Corp Finance. 2015 Jun 1;32:327–46.

[32] Barnea A, Rubin A. Corporate Social Responsibility as a Conflict Between Shareholders. J Bus Ethics. 2010 Nov 1;97(1):71–86.

[33] Does CEO emotion matter? CEO affectivity and corporate social responsibility—Wang—2023—Strategic Management Journal—Wiley Online Library [Internet]. [cited 2024 Jan 18]. Available from: https://onlinelibrary.wiley.com/doi/abs/10.1002/smj.3474

[34] Du X, Jian W, Zeng Q, Du Y. Corporate Environmental Responsibility in Polluting Industries: Does Religion Matter? J Bus Ethics. 2014 Oct 1;124(3):485–507.

[35] Kollmuss A, Agyeman J. Mind the Gap: Why do people act environmentally and what are the barriers to pro-environmental behavior? Environ Educ Res. 2002 Aug 1;8(3):239–60.

[36] Chen T, Dong H, Lin C. Institutional shareholders and corporate social responsibility. J Financ Econ. 2020 Feb 1;135(2):483–504.

[37] Kim JB, Li Y, Zhang L. Corporate tax avoidance and stock price crash risk: Firm-level analysis. J Financ Econ. 2011 Jun 1;100(3):639–62.

[38] Dhaliwal DS, Li OZ, Tsang A, Yang YG. Voluntary Nonfinancial Disclosure and the Cost of Equity Capital: The Initiation of Corporate Social Responsibility Reporting. Account Rev. 2011;86(1):59–100.

[39] Gull AA, Hussain N, Khan SA, Khan Z, Saeed A. Governing Corporate Social Responsibility Decoupling: The Effect of the Governance Committee on Corporate Social Responsibility Decoupling. J Bus Ethics. 2023 Jun;185(2):349–74.

[40] Zaid MAA, Abuhijleh STF, Pucheta‐Martínez MC. Ownership structure, stakeholder engagement, and corporate social responsibility policies: The moderating effect of board independence. Corp Soc Responsib Environ Manag. 2020 May;27(3):1344–60.

[41] Mizuno M. Institutional Investors, Corporate Governance and Firm Performance in Japan. Pac Econ Rev. 2010;15(5):653–65.

[42] Healy PM, Palepu KG. Information asymmetry, corporate disclosure, and the capital markets: A review of the empirical disclosure literature. J Account Econ. 2001 Sep 1;31(1):405–40.

[43] Hao DY, Qi GY, Wang J. Corporate Social Responsibility, Internal Controls, and Stock Price Crash Risk: The Chinese Stock Market. Sustainability. 2018 May;10(5):1675.

[44] Huang C, Ho KC. Does the presence of executives with a legal background affect stock price crash risk? Corp Gov Int Rev. 2023;31(1):55–82.

[45] Scherer AG, Palazzo G. The New Political Role of Business in a Globalized World: A Review of a New Perspective on CSR and its Implications for the Firm, Governance, and Democracy. J Manag Stud. 2011;48(4):899–931.

[46] Tang P, Yang S, Yang S. How to design corporate governance structures to enhance corporate social responsibility in China’s mining state-owned enterprises? Resour Policy. 2020 Jun 1;66:101619.

[47] Lin KJ, Lu X, Zhang J, Zheng Y. State-owned enterprises in China: A review of 40 years of research and practice. China J Account Res. 2020 Mar 1;13(1):31–55.

[48] Megginson WL, Netter JM. From State to Market: A Survey of Empirical Studies on Privatization. J Econ Lit. 2001 Jun;39(2):321–89.

[49] Xu N, Li X, Yuan Q, Chan KC. Excess perks and stock price crash risk: Evidence from China. J Corp Finance. 2014 Apr 1;25:419–34.

[50] Tewari S, Bhattacharya B. Financial resources, corporate social responsibility, and ownership type: Evidence from India. Asia Pac J Manag. 2023 Sep;40(3):1093–132.

[51] Córdoba-Pachón JR, Garde-Sánchez R, Rodríguez-Bolívar MP. A Systemic View of Corporate Social Responsibility (CSR) in State-Owned Enterprises (SOEs). Knowl Process Manag. 2014;21(3):206–19.

[52] Chan MC, Watson J, Woodliff D. Corporate Governance Quality and CSR Disclosures. J Bus Ethics. 2014 Nov 1;125(1):59–73.

[53] Hutton AP, Marcus AJ, Tehranian H. Opaque financial reports, R2, and crash risk. J Financ Econ. 2009 Oct 1;94(1):67–86.

[54] Wang H, Qian C. Corporate Philanthropy and Corporate Financial Performance: The Roles of Stakeholder Response and Political Access. Acad Manage J. 2011 Dec;54(6):1159–81.

[55] Jiraporn P, Jiraporn N, Boeprasert A, Chang K. Does Corporate Social Responsibility (CSR) Improve Credit Ratings? Evidence from Geographic Identification. Financ Manag. 2014;43(3):505–31.

[56] Dumitrescu A, Zakriya M. Stakeholders and the stock price crash risk: What matters in corporate social performance? J Corp Finance. 2021 Apr 1;67:101871.

[57] Ben-Nasr H, Ghouma H. Employee welfare and stock price crash risk. J Corp Finance. 2018 Feb 1;48:700–25.

[58] van der Laan Smith J, Adhikari A, Tondkar RH. Exploring differences in social disclosures internationally: A stakeholder perspective. J Account Public Policy. 2005 Mar 1;24(2):123–51.

[59] Oh WY, Chang YK, Martynov A. The Effect of Ownership Structure on Corporate Social Responsibility: Empirical Evidence from Korea. J Bus Ethics. 2011 Dec 1;104(2):283–97.

[60] Lopatta K, Jaeschke R, Chen C. Stakeholder Engagement and Corporate Social Responsibility (CSR) Performance: International Evidence. Corp Soc Responsib Environ Manag. 2017;24(3):199–209.

[61] Amran A, Devi SS. The impact of government and foreign affiliate influence on corporate social reporting. Manag Audit J. 2008;23(4):386–404.

[62] Young S, Thyil V. Corporate Social Responsibility and Corporate Governance: Role of Context in International Settings. J Bus Ethics. 2014 Jun 1;122(1):1–24.

[63] Ayyagari M, Dau LA, Spencer J. Strategic Responses to FDI in Emerging Markets: Are Core Members More Responsive than Peripheral Members of Business Groups? Acad Manage J. 2015 Dec;58(6):1869–94.

[64] Jain T, Jamali D. Looking Inside the Black Box: The Effect of Corporate Governance on Corporate Social Responsibility. Corp Gov Int Rev. 2016;24(3):253–73. doi: 10.1111/corg.12154

[65] Ghoul SE, Guedhami O, Kim Y. Country-level institutions, firm value, and the role of corporate social responsibility initiatives. J Int Bus Stud. 2017 Apr 1;48(3):360–85.

[66] Dunbar CG, Li ZF, Shi Y. Corporate Social (Ir)responsibility and Firm Risk: The Role of Corporate Governance [Internet]. Rochester, NY; 2021 [cited 2024 Jan 19]. Available from: https://papers.ssrn.com/abstract=3791594

[67] Dai J, Lu C, Qi J. Corporate social responsibility disclosure and stock price crash risk: Evidence from China. Sustainability. 2019;11(2):448.

